# *TLR10* and *NFKBIA* contributed to the risk of hip osteoarthritis: systematic evaluation based on Han Chinese population

**DOI:** 10.1038/s41598-018-28597-2

**Published:** 2018-07-06

**Authors:** Hongtao Tang, Zhenzhen Cheng, Wenlong Ma, Youwen Liu, Zhaofang Tong, Ruibo Sun, Hongliang Liu

**Affiliations:** 1 Department of Hip Injury and Disease, Luoyang Orthopedic Hospital of Henan Province, Luoyang, Henan China; 2 Department of Ankle and Disease Injury and Disease, Luoyang Orthopedic Hospital of Henan Province, Luoyang, Henan China; 3 Department of Trauma, Honghui Hospital,Xi’an Jiaotong University, Xi’an, Shaanxi China

## Abstract

Multiple lines of evidence have confirmed the importance of genetic factors for hip osteoarthritis (HOA). Our study aimed to investigate the associations of *TLR10* and *NFKBIA* with respect to the HOA risk in Han Chinese individuals. A total of 1,043 HOA patients and 2,664 controls were recruited. Then, 23 tag single-nucleotide polymorphisms (SNPs) in the *TLR10* and *NFKBIA* genes were selected for genotyping. Genetic association analyses were conducted in both single-marker and haplotype-based ways. Gene by gene, two-way interactions were analysed using a case-only method. Multiple bioinformatics tools were utilised to examine the potential functional significance of the SNPs. Two significant SNPs, rs11096957 (OR = 1.26, *P* = 1.35 × 10^−5^) and rs2273650 (OR = 1.2, *P* = 1.57 × 10^−3^), were significantly associated with HOA risk. Rs11096957 was also associated with the severity of the HOA. Bioinformatics analysis indicated that the allele T of rs2273650 would create new miRNA/SNP target duplexes, which suggests that rs2273650 could alter the *NFKBIA* expression by affecting the miRNA/SNP target duplexes. Our study identified significant association signals from *NFKBIA* with HOA for the first time, and it also confirmed the contribution of *TLR10* to the HOA risk. These findings would provide clues for identifying individuals at high risk of HOA.

## Introduction

Osteoarthritis (OA) is a chronic multiple degenerative joint disease that affects an estimated 10% of men and 18% of women over 60 years of age^1^. OA is mainly divided into knee OA (KOA) and hip OA (HOA), and the main pathological manifestations are degeneration of the articular cartilage, osteophyte formation and/or subchondral bone sclerosis. The clinical manifestations include stiffness and pain in the joints that can even result in the loss of joint function and disability, which has brought a heavy burden to society and family^2^. Similar to other chronic and complex diseases, OA is also affected by environmental and genetic factors^3^. The incidence of OA can be influenced by age and gender^4^, and multiple genetic studies have confirmed the importance of the genetic factors. Twin and family studies have proved that the heritability of OA in women is 39–65%^5^. Among them, KOA is 40%, and HOA is 60%^5^. Hence, it is necessary to find the genes that are responsible for susceptibility to OA through candidate gene studies. Combined with a recently published large scale GWAS paper^6^, a total number of 30 loci have been identified and validated for conferring the risk to OA. However, all of these established loci could only explain 26.3% trait variance of OA^6^.

Toll-like receptors (TLR) constitute a family of transmembrane proteins that are expressed in important immune system cells. They play an important role in innate immunity and the induction of inflammatory signalling pathways^7^. There are 10 TLR family members (TLR1-10) in human beings^8^. Thus far, *TLR* gene polymorphisms have been reported to be associated with OA. Through a two-stage case-control study, researchers have shown that a single nucleotide polymorphism (SNP) in the promoter region of *TLR-3* is associated with *TLR-3* gene expression and susceptibility to KOA^9–11^. Another two case-control studies have found that *TLR-9* gene polymorphisms have significant roles in KOA in both the Chinese and Turkish population^12,13^. In addition, several studies have demonstrated that the TLR family mRNA transcription and protein expression level is much higher in OA synovial and cartilage than in normal cartilage^14,15^. For animal experiments, the knockout of *TLR-4* resulted in a less severe phenotype in a mouse model of arthritis^16^, which indicates that the overexpression of the *TLR-4* gene could produce an excessive inflammatory response and result in OA. Therefore, the TLR family could be involved in the occurrence and development of OA. In fact, TLR activation causes increased expression of pro-inflammatory cytokines that are regulated by signalling pathways that involve nuclear factor kB (NF-kB) transcription factors^17,18^, which have been identified to play a significant role in OA^19^. However, among the TLR family, *TLR-10* is clustered together with *TLR-1* and *TLR-6* in the region of 4p14^20^ and is the only member of the TLR family that has an anti-inflammatory effect by inhibiting NFkB signalling^21,22^. NF-kB is held in the cytoplasm in an inactive state complexed with IkBa, an inhibitory protein that is encoded by the *NFKBIA* gene in the genomic region of 14q13.2 in resting cells^23^. IkBa is phosphorylated by inducible expression of IkB kinases (IKKs), which mediates IkBa degradation to result in activated NF-kB translocation to the nucleus to initiate inflammation-related gene transcription^24^. Thus, it is reasonable to hypothesise that common variants of *NFKB1A* could potentially regulate NF-kB signalling and alter cytokine profiles, which would lead to inflammatory responses in some susceptible individuals.

Recently, a study in the Croatian population that involved 500 OA patients and 597 controls reported that a single-nucleotide polymorphism (SNP) rs11096957 in *TLR-10* is significantly associated with predisposition to HOA (*P* = 0.04, OR = 1.41, 95% CI = 1.02–1.94)^21^. Although the relationship of the *NFKBIA* gene with KOA was evaluated in European populations that consisted of 189 KOA cases and 197 healthy controls, the SNP rs8904 was identified to be marginally associated with KOA only in females (*P* = 0.02)^25^. Until now, they are the only two studies that report an association between *TLR-10* and *NFKBIA* correlated with OA in European populations, and these results suggest that *TLR-10* and *NFKBIA* could be involved in the pathogenesis of HOA. However, given that different ethnic populations could exhibit genetic heterogeneity of OA, the replications of the study using large-scale samples from other different populations are needed to confirm these results. Although several previous published GWASs have included both *TLR10* and *NFKBIA*^26–28^, none SNPs of the two loci have been reported to achieve genome-wide significance. In addition, there is no research that regards *TLR-10* and *NFKBIA* in terms of the risk of HOA in the Han Chinese population. Thus, the present case-control study aims to investigate the associations of *TLR-10* and *NFKBIA* with HOA risk in Han Chinese individuals, which would potentially shed light on the underlying pathological mechanisms of OA.

## Methods

### Study subjects

In our study, a total of 3,707 study subjects comprised of 1,043 HOA patients and 2,664 controls were recruited from Luoyang Orthopedic Hospital of Henan Province. All of the subjects were random unrelated Han Chinese individuals. The diagnosis of HOA was based on the criteria of the American College of Rheumatology, and all of the controls had no signs or symptoms of arthritis or joint disease (pain, swelling, tenderness, or movement restriction). The HOA patients were confirmed by clinical examination and radiographic inspection. A questionnaire was used to collect demographic characteristics from subjects with regard to general information, smoking, drinking, occupations, sports activities, previous hip injuries and family history of OA and other diseases (Table 1). Only patients with a score of 2 or more (based on the Kellgren-Lawrence (K-L) grading standard) were included in the present study. The healthy control subjects had no symptoms of arthritis or any other joint-related disorders and had no family history of OA or other rheumatic diseases. Study subjects were excluded if they had inflammatory arthritis (rheumatoid, polyarthritic or autoimmune disease), post-traumatic or post-septic arthritis, and skeletal or developmental dysplasia. Further, all of the subjects in both groups were free of systemic or organic diseases. This study was performed according to the ethical guidelines of the Declaration of Helsinki (version 2002) and was approved by the Luoyang Orthopedic Hospital of Henan Province. Informed consent forms were obtained from all subjects.

**Table 1:** Characteristic information of study subjects.

	HOA patients (N = 1,043)	controls (N = 2,664)	statistics	*P*
Age, mean ± sd	61.8 ± 8.0	60.7 ± 8.1	t = 3.97	7.41 × 10^−5^
BMI, mean ± sd	26.2 ± 1.5	25.8 ± 1.5	t = 7.15	1.23 × 10^−12^
Gender (%)				
Male	497 (48)	1287 (48)		
Female	546 (52)	1377 (52)	χ^2^ = 0.11	0.7452
Smoking (%)				
Yes	248 (24)	618 (23)		
No	795 (76)	2046 (77)	χ^2^ = 0.11	0.7401
Drinking alcohol(%)				
Yes	307 (29)	815 (31)		
No	736 (71)	1849 (69)	χ^2^ = 0.42	0.5152
Occupation (%)				
Managerial	446 (43)	1162 (44)		
Non-managerial	597 (57)	1502 (56)	χ^2^ = 0.19	0.6623
Activity (%)				
Inactive	400 (38)	1042 (39)		
Less active	366 (35)	981 (37)		
More active	277 (27)	641 (24)	χ^2^ = 2.62	0.2701
KL grading scale (%)				
KL-2	510 (49)	—		
KL-3	388 (37)	—		
KL-4	145 (14)	—	—	—

### SNP Selection and Genotyping

Tag SNPs covered the gene regions of *TLR10* and *NFKBIA* and were selected for genotyping based on the 1,000 genome data of Chinese Han populations. Minor allele frequency (MAF) > 0.01 and r^2^ > 0.6 were utilised as criteria for tagging. A total of 23 tag SNPs, 14 from *TLR10* and 9 from *NFKBIA*, were selected for genotyping. Genomic DNA was extracted from peripheral blood leukocytes according to the manufacturer’s protocol (Genomic DNA kit, Axygen Scientific Inc., California, USA). Genotyping was performed for all SNPs using the Sequenom Mass ARRAY RS1000 system (Sequenom, San Diego, California, USA). The results were processed using Typer Analyzer software (Sequenom)^29^, and the genotype data were generated from the samples. To ensure the accuracy of the genotyping, we have randomly chosen 5% of our study subjects and repeated the genotyping process for them. The concordance rate of this process was 100%, which indicates that the genotyping results of our study were reliable.

### Statistical Analyses

MAFs were calculated, and Hardy-Weinberg equilibrium was tested for all of the genotyped SNPs. Logistic models were fitted for each SNP, with age and BMI added as covariates to investigate the potential genetic associations. Haplotypic association analyses were also performed. The analyses above were performed by the genetic analysis software Plink^30^. Genomic control was conducted to detect and correct the potential inflation of significance caused by underlying population stratification^31,32^, and a null distribution of the inflation factor λ was created by 10,000 bootstrapping. Multiple comparisons were corrected by Bonferroni corrections, and thus, the threshold of the *P* values used for single marker-based association analyses were 0.05/23 ≈ 0.002. The associations of targeted SNPs with disease severity measurements of K-L grade scale were evaluated by χ^2^ tests. In addition, because of the potential biological connections between *TLR10* and *NFKBIA*, there could be a potential effect of gene-by-gene interactions on the risk of HOA from both genes. We have tested this two-locus interaction by conducting case-only tests^33^ for all SNP pairs between *TLR10* and *NFKBIA* using Plink.

### Bioinformatics Analyses

Bioinformatics analyses were performed to investigate the potential functional significance of the targeted SNPs, and two types of tools were utilised. For non-synonymous SNPs, we examined their functional consequences on the gene products using Polyphen2^34^ and SIFT^35^. In addition, RegulomeDB^36^, which evaluated the evidence for the functional significance of SNPs using ENCODE data, was also utilised. RegulomeDB assigned a score that ranged from 1 to 7 for each SNP to indicate its functional significance. In addition, we examined the effect of targeted SNPs on miRNA-mediated gene repression using PolymiRTS (http://compbio.uthsc.edu/miRSNP/home.php)^37^.

### Expression quantitative trait loci (eQTL) analyses

We extracted eQTL data for ~40 human tissues of targeted SNPs from the GTEx database (https://www.gtexportal.org/home/)^38^. Data on the gene expression for different genotypes were extracted and compared. Significant SNPs with eQTL effects were reported.

## Results

### Genetic associations of *TLR10* and *NFKBIA* with HOA

As shown in Table 1, the age and BMI were distributed differently between the cases and control. No significant differences could be identified for the other individuals and environmental factors, including the gender, smoking status, alcohol drinking, occupation type, and activity. Two significant SNPs, rs11096957 (OR = 1.26, *P* = 1.35 × 10^−5^) and rs2273650 (OR = 1.2, *P* = 1.57 × 10^−3^), were identified to be significantly associated with the disease status of HOA after adjusting for the effects of age and BMI (Table 2). The SNP rs11096957 is a non-synonymous SNP that is located in the exonic region of the gene *TLR10*. The SNP rs2273650 is located at the 3′ untranslated region (UTR) of the gene *NFKBIA*. The mean statistics for the 21 non-significance tests was 0.47 with a 95% confidence interval [0.37, 0.57] (Supplemental Fig. S1). This finding indicated that no significant inflation of the test statistics could be obtained from our single-marker-based association analyses. Therefore, there were very limited effects of population stratification for this study. This significant hit of the gene *NFKBIA* was validated and replicated in haplotypic association analyses. Linkage disequilibrium (LD) blocks were constructed for both genes separately, and the haplotypes were tested for their associations with HOA (Supplemental Figs S2 and S3). A two-SNP haplotype, rs2273650–rs8904, of gene *NFKBIA* was identified to be significantly associated with HOA (Table 3).

**Table 2:** Results of single marker based association analyses for SNPs of *NFKBIA* and *TLR10*.

CHR	SNP	POS	A1	Gene	Function	MAF	HWE	OR	*P*
4	rs78826685	38773942	C	*TLR10*	untranslated-3	0.06	0.72	1.07	0.55
4	rs9715769	38774489	C	*TLR10*	untranslated-3	0.43	0.43	0.99	0.82
4	rs11466658	38775639	A	*TLR10*	missense	0.08	0.59	0.95	0.63
4	rs11466655	38776070	T	*TLR10*	missense	0.22	0.82	0.98	0.70
4	rs145872263	38776355	C	*TLR10*	missense	0.07	0.16	1.05	0.66
4	**rs11096957**	**38776491**	**T**	***TLR10***	**missense**	**0**.**39**	**0**.**15**	**1**.**26**	**1**.**35 × 10**^**−5**^
4	rs10856838	38777173	T	*TLR10*	coding-synon	0.19	0.71	1.04	0.55
4	rs10002420	38779213	A	*TLR10*	intron	0.10	1.00	1.06	0.50
4	rs4130727	38781113	G	*TLR10*	intron	0.09	1.00	0.98	0.80
4	rs9997988	38782843	C	*TLR10*	intron	0.15	0.94	1.02	0.82
4	rs59672618	38783171	C	*TLR10*	intron	0.09	1.00	0.93	0.42
4	rs79030744	38783588	C	*TLR10*	intron	0.14	0.19	0.93	0.34
4	rs10031946	38783840	G	*TLR10*	intron	0.08	0.49	1.01	0.88
4	rs11725309	38783848	T	*TLR10*	intron	0.49	0.85	0.95	0.36
14	**rs2273650**	**35870798**	**T**	***NFKBIA***	**untranslated-3**	**0**.**28**	**0**.**34**	**1**.**20**	**1**.**57 × 10**^**−3**^
14	rs8904	35871217	A	*NFKBIA*	untranslated-3	0.40	1.00	1.01	0.88
14	rs1022714	35871407	A	*NFKBIA*	intron	0.23	0.96	0.97	0.67
14	rs2233416	35872765	A	*NFKBIA*	intron	0.07	0.52	1.07	0.52
14	rs2233415	35872792	A	*NFKBIA*	intron	0.35	0.76	1.02	0.78
14	rs1050851	35872926	A	*NFKBIA*	coding-synon	0.03	0.47	0.90	0.52
14	rs2233411	35873567	T	*NFKBIA*	intron	0.11	0.76	0.97	0.70
14	rs1957106	35873770	A	*NFKBIA*	coding-synon	0.23	0.44	1.00	0.96
14	rs2273651	35873916	T	*NFKBIA*	untranslated-5	0.04	0.25	0.93	0.58

CHR: chromosome; POS: position; A1: tested allele; MAF: minor allele frequency; HWE: Hardy-Weinberg equilibrium test. Significant results were highlighted in bold.

**Table 3:** Results of haplotype-based association analyses.

Gene	SNPs	χ^2^	DF	*P*
***NFKBIA***	**rs2273650–rs8904**	**20**.**55**	**2**	**3**.**45 × 10**^**−5**^
*TLR10*	rs11466658–rs11466655	0.70	2	0.70
*TLR10*	rs10856838–rs10002420	0.44	2	0.80
*TLR10*	rs9997988–rs59672618	3.67	2	0.16

DF: degree of freedom. Significant result was highlighted in bold.

### Associations of rs11096957 with the severity of HOA

Both significant SNPs identified in the single-marker-based association analyses, rs11096957 and rs2273650, were tested for their associations with the severity of HOA in the patient samples. Rs11096957 from gene *TLR10* was found to be significantly associated with the severity of HOA as measured by KL grade scaling (Table 4). The T allele of rs11096957 was a significant indicator of the severity of HOA in our samples, and the pattern was very clear. All of the KL-4 grade HOA patients had a genotype of TT for rs11096957, while no patients of HOA with the KL-2 grade had a genotype of TT.

**Table 4:** Association analyses of targeted SNPs with KL grading scale of HOA patients.

SNP	KL Grading Scale (%)				χ^2^	*P*
rs2273650		TT (N = 101)	CT (N = 430)	CC (N = 512)		
*NFKBIA*	KL-2	50 (50)	210 (49)	250 (49)		
	KL-3	38 (38)	160 (37)	190 (37)		
	KL-4	13 (12)	60 (14)	72 (14)	0.1	0.9987
rs11096957		TT (N = 201)	GT (N = 486)	GG (N = 356)		
*TLR10*	KL-2	0 (0)	154 (32)	356 (100)		
	KL-3	56 (28)	332 (68)	0 (0)		
	KL-4	145 (72)	0 (0)	0 (0)	1188.9	<0.0001

### Epistasis analyses

Case-only tests were performed for all SNP pairs between *TLR10* and *NFKBIA*. A total of 126 tests were conducted (14 × 9). No significant SNP pairs were identified after applying the Bonferroni correction (the threshold of the *P* value is 0.05/126 ≈ 4 × 10^−4^). The most significant SNP pair was rs11096957 (*TLR10*) and rs8904 (*NFKBIA*) with *P* = 0.0165 (Supplemental Table S1).

### Functional consequences of the selected SNPs

We obtained the functional consequences for the significant non-synonymous SNP rs11096957 of *TLR10* (Table 5). For rs11096957, both Polyphen2 and SIFT have classified it as an SNP with a functional consequence (“possibly damaging” for Polyphen2 and “damaging” for SIFT) for the protein encoded by *TLR10*. In addition, the minor allele of the significant SNP from *NFKBIA* was found to create new binding sites for multiple miRNAs, which could mediate the down-regulation of *NFKBIA* expression. The RegulomeDB scores for rs11096957 and rs2273650 were 7 and 4, respectively, which indicates that the evidence of functional significance for both SNPs was very limited.

**Table 5:** Functional consequences for candidate SNPs based on bioinformatics analyses.

Chr	Position	SNP	RegSc	Polyphen2	SIFT	PolymiRTS
4	38776490	rs11096957	7	possibly damaging	DAMAGING	—
14	35870797	rs2273650	4	—	—	The derived allele creates new miRNA site: hsa-miR-4266/ hsa-miR-4695-5p/ hsa-miR-4729/ hsa-miR-4779

RegSc: Score from RegulomeDB.

### Analyses of eQTL

**Figure 1:**
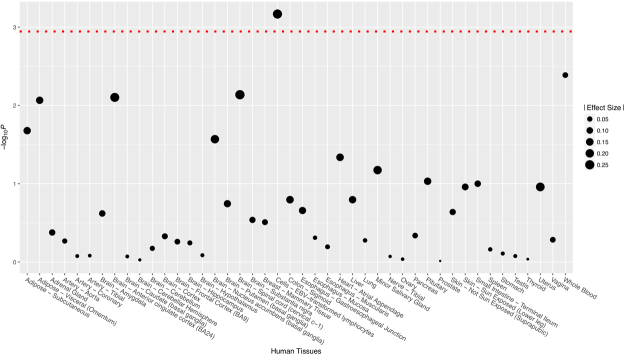
The eQTL pattern of SNP rs11096957 for *TLR10* in 45 human tissues. The *P* value threshold is indicated by the red dotted line.

We extracted eQTL data of 44 human tissues from GTEx for rs1109695 (no data can be obtained for rs2273650 due to its low MAF in Europeans). A significant signal for eQTL was observed only from cells of EBV-transformed lymphocytes (Fig. 1).

## Discussion

In this study, we gained evidence that supports the roles that *TLR10* plays in the onset and development of HOA. We identified a significant non-synonymous SNP, rs11096957, which was significantly associated with the disease status of HOA. Although a previous candidate gene-based study obtained similar results with a sample from European populations^21^, our findings can serve as a successful replication of this previous study in the Chinese Han population. Compared to the previous study, which focused only on the association with the disease status of OA, we also examined the association between the severity of HOA and rs11096957. Our findings showed that rs11096957 could serve as a significant indicator for the severity of HOA, when measured by the KL grade scale. A dosage-dependent pattern for the T allele of rs11096957 can be found to be associated with the severity of HOA. In addition, according to the results of bioinformatics analyses, the minor allele of rs11096957 can significantly alter the structure of the protein encoded by *TLR10* and in turn affect its function fundamentally. On the other hand, eQTL analyses offered us only limited evidence for the role of rs11096957 on the gene expression level of *TLR10* (1 significant hit out of 45 human tissues). Combined with all of this evidence from different aspects, we believe that the SNP rs11096957 was susceptible to HOA with a true functional effect by disrupting the structure of the protein encoded by *TLR10*. The association signal identified from our study could be more than solely a statistical association but could also have a functional effect and a representation of biological and pathological mechanisms.

Another significant hit was obtained for the gene *NFKBIA*. The SNP rs2273650 is located at the 3′ untranslated region of *NFKBIA*, and thus, unlike exonic variants, it has nothing to do with the protein structure. We determined that the T allele of this SNP could increase the risk of HOA by approximately 20%, although no significant association was identified between this SNP and the severity of HOA in our case samples. To the best of our knowledge, our study was the first study to identify a significant association signal for the SNP rs2273650. A previous study that was based on European women determined that another SNP of *NFKBIA*, rs8904, is associated with OA^25^. The SNP rs2273650 was not genotyped in that study because of its low MAF in the European population (MAF = 0.005 in the European population based on data from the 1000 genomes project). Interestingly, both rs2273650 and rs8904 were genotyped in our study, and the two SNPs were in strong LD. Given that it is not sufficient to draw conclusions from limited SNPs analyses^39–41^, we performed haplotype analyses, which indicated a similar pattern with single marker-based associations. The haplotype formed by these two SNPs was significantly associated with HOA disease status. However, unlike rs2273650, there is very limited evidence to indicate the functional significance of rs8904, and it is probably only a surrogate of some underlying SNP that has a functional effect. Functional analyses have shown that the allele T of the SNP rs2273650 could create some new binding sites for four types of miRNA and in turn repress the gene expression of *NFKBIA*. The gene *NFKBIA* encoded the protein IkBa, and this protein can serve as an inhibitory protein for NF-kB, which was an important factor for the activation of inflammation-related gene transcription^23^. A reasonable mechanism is that the allele T of the SNP rs2273650 could repress the expression of *NFKBIA* and in turn activate NF-kB translocation to the nucleus to initiate inflammation-related gene transcription. The functional direction of the T allele in the regulation of *NFKBIA* expression is the same as its contribution to the risk of HOA in our sample. Combined with all of this evidence gained in this study and the previous study, we could induce that rs2273650 might not be the only SNP that has an effect on the onset and development of HOA. Multiple variants clustered at the 3′ untranslated region of *NFKBIA* might affect the risk of OA in an independent way, and an ethnicity-specific pattern was expected to be observed due to the differences in the LD structures of the local genomic regions.

*TLR10* is the only member of the Toll-like receptor that has an anti-inflammatory effect by inhibiting the NFkB signalling^21,22^. These biological connections between *TLR10* and IkBa might be reflected at the level of gene-to-gene interactions. However, our two-way interaction analyses failed to detect any significant epistasis signals between *NFKBIA* and *TLR10*. Nevertheless, it is too early to make any conclusions based on this result. The lack of statistical power and the low information coverage of SNPs on each gene might explain this negative finding.

Our study suffered from several limitations. First, cautions must be taken to explain our data, especially when most of the functional analyses were conducted with a “dry lab” without being validated by our own experimental data. GTEx eQTL data were mainly based on the European population and thus might offer limited reference for our study, which was based on the Chinese Han population. In addition, the role of the T allele of rs2273650 in the miRNA binding sites was totally predicted based on bioinformatics tools, and no experimental data based on double-Luciferase reporter assay technology from our study were reported to validate this finding. Second, we have tried our best to restrict population stratification when recruiting subjects by restricting the study subjects with stable living area^42,43^, but the potential population stratification could not be completely ruled out. Third, the significant hit of *NFKBIA* has never been replicated in any population, and therefore, we cannot rule out its chance of being a false positive. In this study, we have applied candidate gene based study design which was a cost-effective design for fine mapping or replication of well-established loci. However, it had poor performance in novel loci identifications partly due to its poor records in reproducibility. Therefore, replication studies based on different populations are still needed in future.

In summary, in this study, we identified significant association signals from *NFKBIA* and *TLR1* with HOA in a large Chinese Han-based sample. Two SNPs, rs11096957 and rs2273650, were determined to contribute to the risk of HOA. Bioinformatics analyses have provided supportive evidence for the role of functional significance for both SNPs.

## Electronic supplementary material

Supplemental materials
