# Supplementary material for: TLR10 and NFKBIA contributed to the risk of hip osteoarthritis: systematic evaluation based on Han Chinese population
Source: Sci Rep. 2018 Jul 6;8:10243. doi: 10.1038/s41598-018-28597-2 (PMC6035240; doi:10.1038/s41598-018-28597-2)
Supplement: Supplementary file 1 — Supplemental materials [file 41598_2018_28597_MOESM1_ESM.docx]

***Title***: *TLR10* and *NFKBIA* contributed to the risk of hip osteoarthritis: systematic evaluation based on Han Chinese population

***Author names and affiliations***: Hongtao Tang ^1*^, Zhenzhen Cheng ^2*^, Wenlong Ma ^1^, Youwen Liu ^1^, Zhaofang Tong ^1^, Ruibo Sun ^1^ and Hongliang Liu ^3^

^1^ Department of Hip Injury and Disease, Luoyang Orthopedic Hospital of Henan Province, Luoyang, Henan, China;

^2^ Department of Ankle and Disease Injury and Disease, Luoyang Orthopedic Hospital of Henan Province, Luoyang, Henan, China;

^3^ Department of Trauma, Honghui Hospital，Xi’an Jiaotong University, Xi'an, Shaanxi, China.

These authors contributed equally to the work.

***Corresponding Author***:

Hongliang Liu, M.D. & Ph.D., Department of Trauma, Honghui Hospital, Xi’an Jiaotong University Health Science Center, No.555, Youyi East Road, Xi'an, Shaanxi, China, 710054.

Tel: 86-29-88418023; Fax: 86-29-62818386; E-mail: osteohlliu@163.com

Supplemental Table S1. Results for epistasis analyses.

| CHR1 | SNP1 | CHR2 | SNP2 | STAT | *P* |
| --- | --- | --- | --- | --- | --- |
| 4 | rs11096957 | 14 | rs8904 | 5.7510 | 0.0165 |
| 4 | rs10856838 | 14 | rs1957106 | 4.3010 | 0.0381 |
| 4 | rs4130727 | 14 | rs2233415 | 4.0360 | 0.0445 |
| 4 | rs79030744 | 14 | rs1022714 | 3.9900 | 0.0458 |
| 4 | rs78826685 | 14 | rs2273651 | 3.6400 | 0.0564 |
| 4 | rs145872263 | 14 | rs8904 | 3.2510 | 0.0714 |
| 4 | rs9997988 | 14 | rs2273650 | 3.1470 | 0.0761 |
| 4 | rs10856838 | 14 | rs2233416 | 2.9560 | 0.0856 |
| 4 | rs10856838 | 14 | rs2233411 | 2.7420 | 0.0977 |
| 4 | rs9715769 | 14 | rs2233416 | 2.1780 | 0.1400 |
| 4 | rs145872263 | 14 | rs1022714 | 2.1370 | 0.1438 |
| 4 | rs4130727 | 14 | rs2233411 | 1.9370 | 0.1640 |
| 4 | rs10002420 | 14 | rs1050851 | 1.9270 | 0.1651 |
| 4 | rs11096957 | 14 | rs1022714 | 1.8290 | 0.1763 |
| 4 | rs4130727 | 14 | rs2233416 | 1.7570 | 0.1850 |
| 4 | rs4130727 | 14 | rs1022714 | 1.7510 | 0.1858 |
| 4 | rs9715769 | 14 | rs8904 | 1.6750 | 0.1956 |
| 4 | rs11096957 | 14 | rs2233411 | 1.6730 | 0.1959 |
| 4 | rs9715769 | 14 | rs1050851 | 1.6520 | 0.1987 |
| 4 | rs10002420 | 14 | rs2233415 | 1.6160 | 0.2036 |
| 4 | rs4130727 | 14 | rs2273650 | 1.6040 | 0.2054 |
| 4 | rs11466655 | 14 | rs8904 | 1.4150 | 0.2342 |
| 4 | rs79030744 | 14 | rs2233411 | 1.3740 | 0.2411 |
| 4 | rs9997988 | 14 | rs1957106 | 1.2810 | 0.2578 |
| 4 | rs4130727 | 14 | rs1050851 | 1.2700 | 0.2598 |
| 4 | rs11096957 | 14 | rs2273650 | 1.2000 | 0.2733 |
| 4 | rs59672618 | 14 | rs2273650 | 1.1840 | 0.2765 |
| 4 | rs10031946 | 14 | rs2233416 | 1.1720 | 0.2791 |
| 4 | rs10002420 | 14 | rs2233411 | 1.1390 | 0.2858 |
| 4 | rs11466658 | 14 | rs8904 | 1.1270 | 0.2885 |
| 4 | rs4130727 | 14 | rs8904 | 1.1220 | 0.2896 |
| 4 | rs9997988 | 14 | rs2233415 | 1.0840 | 0.2979 |
| 4 | rs59672618 | 14 | rs1050851 | 1.0600 | 0.3032 |
| 4 | rs145872263 | 14 | rs2233416 | 0.9852 | 0.3209 |
| 4 | rs9997988 | 14 | rs2273651 | 0.9634 | 0.3263 |
| 4 | rs11096957 | 14 | rs1957106 | 0.9556 | 0.3283 |
| 4 | rs11725309 | 14 | rs2273651 | 0.9479 | 0.3302 |
| 4 | rs10002420 | 14 | rs2273650 | 0.9318 | 0.3344 |
| 4 | rs78826685 | 14 | rs2273650 | 0.9124 | 0.3395 |
| 4 | rs78826685 | 14 | rs8904 | 0.8978 | 0.3434 |
| 4 | rs11725309 | 14 | rs2233411 | 0.8897 | 0.3456 |
| 4 | rs10856838 | 14 | rs2273651 | 0.8844 | 0.3470 |
| 4 | rs9997988 | 14 | rs1022714 | 0.8845 | 0.3470 |
| 4 | rs11096957 | 14 | rs2233415 | 0.8700 | 0.3510 |
| 4 | rs11466655 | 14 | rs1022714 | 0.8439 | 0.3583 |
| 4 | rs10856838 | 14 | rs8904 | 0.7994 | 0.3713 |
| 4 | rs78826685 | 14 | rs2233415 | 0.7574 | 0.3841 |
| 4 | rs10002420 | 14 | rs2233416 | 0.7376 | 0.3904 |
| 4 | rs11466655 | 14 | rs2273650 | 0.7111 | 0.3991 |
| 4 | rs59672618 | 14 | rs2233415 | 0.7057 | 0.4009 |
| 4 | rs9997988 | 14 | rs8904 | 0.6904 | 0.4060 |
| 4 | rs10002420 | 14 | rs1957106 | 0.6755 | 0.4111 |
| 4 | rs10856838 | 14 | rs2273650 | 0.6666 | 0.4143 |
| 4 | rs10031946 | 14 | rs1022714 | 0.6569 | 0.4176 |
| 4 | rs59672618 | 14 | rs2233416 | 0.6485 | 0.4207 |
| 4 | rs59672618 | 14 | rs1957106 | 0.6290 | 0.4277 |
| 4 | rs145872263 | 14 | rs2273651 | 0.6208 | 0.4307 |
| 4 | rs11725309 | 14 | rs2233416 | 0.5889 | 0.4428 |
| 4 | rs10031946 | 14 | rs2273651 | 0.5727 | 0.4492 |
| 4 | rs79030744 | 14 | rs8904 | 0.5713 | 0.4498 |
| 4 | rs10002420 | 14 | rs1022714 | 0.5680 | 0.4510 |
| 4 | rs11466658 | 14 | rs1022714 | 0.5135 | 0.4736 |
| 4 | rs11466658 | 14 | rs2233411 | 0.4890 | 0.4844 |
| 4 | rs9715769 | 14 | rs2273651 | 0.4607 | 0.4973 |
| 4 | rs11466655 | 14 | rs1050851 | 0.4563 | 0.4994 |
| 4 | rs9715769 | 14 | rs2273650 | 0.4394 | 0.5074 |
| 4 | rs11725309 | 14 | rs1022714 | 0.4384 | 0.5079 |
| 4 | rs10856838 | 14 | rs1050851 | 0.4265 | 0.5137 |
| 4 | rs10002420 | 14 | rs8904 | 0.4128 | 0.5206 |
| 4 | rs145872263 | 14 | rs1957106 | 0.3907 | 0.5319 |
| 4 | rs11466655 | 14 | rs1957106 | 0.3642 | 0.5462 |
| 4 | rs11096957 | 14 | rs1050851 | 0.3554 | 0.5511 |
| 4 | rs10031946 | 14 | rs2233415 | 0.3489 | 0.5548 |
| 4 | rs9715769 | 14 | rs1957106 | 0.3420 | 0.5587 |
| 4 | rs145872263 | 14 | rs1050851 | 0.3322 | 0.5644 |
| 4 | rs79030744 | 14 | rs2273650 | 0.3290 | 0.5663 |
| 4 | rs11466655 | 14 | rs2233416 | 0.3027 | 0.5822 |
| 4 | rs10002420 | 14 | rs2273651 | 0.2724 | 0.6017 |
| 4 | rs10856838 | 14 | rs2233415 | 0.2511 | 0.6163 |
| 4 | rs145872263 | 14 | rs2233415 | 0.2305 | 0.6312 |
| 4 | rs11466658 | 14 | rs2233415 | 0.2272 | 0.6336 |
| 4 | rs11466658 | 14 | rs2273650 | 0.2266 | 0.6340 |
| 4 | rs11466658 | 14 | rs2233416 | 0.2189 | 0.6399 |
| 4 | rs9715769 | 14 | rs2233415 | 0.2065 | 0.6495 |
| 4 | rs11466658 | 14 | rs1050851 | 0.1887 | 0.6640 |
| 4 | rs11725309 | 14 | rs8904 | 0.1780 | 0.6731 |
| 4 | rs9715769 | 14 | rs1022714 | 0.1552 | 0.6936 |
| 4 | rs79030744 | 14 | rs2233415 | 0.1536 | 0.6951 |
| 4 | rs11466655 | 14 | rs2233415 | 0.1280 | 0.7205 |
| 4 | rs10856838 | 14 | rs1022714 | 0.1222 | 0.7267 |
| 4 | rs11466658 | 14 | rs2273651 | 0.1210 | 0.7279 |
| 4 | rs59672618 | 14 | rs2233411 | 0.1209 | 0.7281 |
| 4 | rs11096957 | 14 | rs2233416 | 0.0911 | 0.7628 |
| 4 | rs4130727 | 14 | rs1957106 | 0.0791 | 0.7785 |
| 4 | rs59672618 | 14 | rs8904 | 0.0768 | 0.7817 |
| 4 | rs78826685 | 14 | rs1050851 | 0.0754 | 0.7836 |
| 4 | rs10031946 | 14 | rs1957106 | 0.0753 | 0.7838 |
| 4 | rs11466655 | 14 | rs2273651 | 0.0751 | 0.7840 |
| 4 | rs79030744 | 14 | rs1050851 | 0.0738 | 0.7859 |
| 4 | rs78826685 | 14 | rs2233416 | 0.0726 | 0.7876 |
| 4 | rs9715769 | 14 | rs2233411 | 0.0714 | 0.7892 |
| 4 | rs78826685 | 14 | rs2233411 | 0.0641 | 0.8001 |
| 4 | rs145872263 | 14 | rs2273650 | 0.0608 | 0.8052 |
| 4 | rs11096957 | 14 | rs2273651 | 0.0545 | 0.8153 |
| 4 | rs11725309 | 14 | rs2233415 | 0.0439 | 0.8341 |
| 4 | rs10031946 | 14 | rs2273650 | 0.0414 | 0.8388 |
| 4 | rs11725309 | 14 | rs1957106 | 0.0412 | 0.8392 |
| 4 | rs78826685 | 14 | rs1022714 | 0.0370 | 0.8475 |
| 4 | rs11725309 | 14 | rs2273650 | 0.0368 | 0.8479 |
| 4 | rs10031946 | 14 | rs1050851 | 0.0316 | 0.8589 |
| 4 | rs11725309 | 14 | rs1050851 | 0.0293 | 0.8641 |
| 4 | rs9997988 | 14 | rs1050851 | 0.0255 | 0.8732 |
| 4 | rs78826685 | 14 | rs1957106 | 0.0236 | 0.8779 |
| 4 | rs145872263 | 14 | rs2233411 | 0.0134 | 0.9077 |
| 4 | rs4130727 | 14 | rs2273651 | 0.0107 | 0.9175 |
| 4 | rs10031946 | 14 | rs2233411 | 0.0058 | 0.9392 |
| 4 | rs59672618 | 14 | rs2273651 | 0.0054 | 0.9415 |
| 4 | rs79030744 | 14 | rs2273651 | 0.0022 | 0.9624 |
| 4 | rs10031946 | 14 | rs8904 | 0.0015 | 0.9686 |
| 4 | rs11466658 | 14 | rs1957106 | 0.0013 | 0.9712 |
| 4 | rs79030744 | 14 | rs2233416 | 0.0010 | 0.9751 |
| 4 | rs9997988 | 14 | rs2233411 | 0.0004 | 0.9849 |
| 4 | rs79030744 | 14 | rs1957106 | 0.0003 | 0.9856 |
| 4 | rs9997988 | 14 | rs2233416 | 0.0001 | 0.9905 |
| 4 | rs59672618 | 14 | rs1022714 | 2.84E-05 | 0.9957 |
| 4 | rs11466655 | 14 | rs2233411 | 6.18E-06 | 0.9980 |


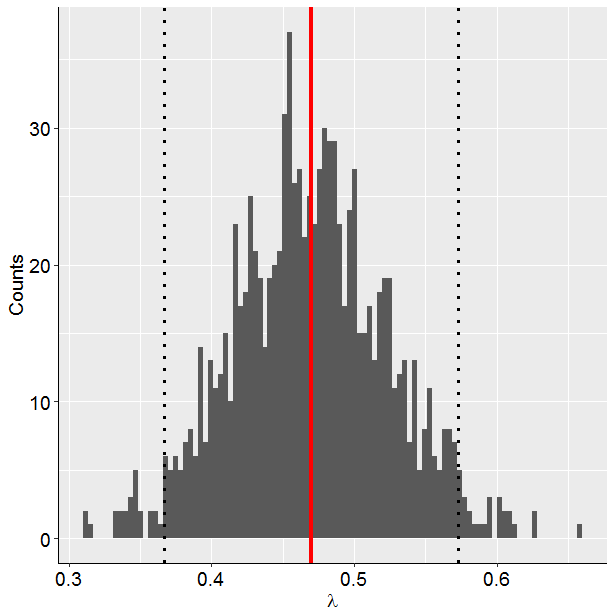


Supplemental Figure S1. Histogram for distribution of inflation factor lambda obtained through 10,000 bootstrapping. Observed value of lambda was indicated in red. 95% confidence intervals were indicated by dotted lines.


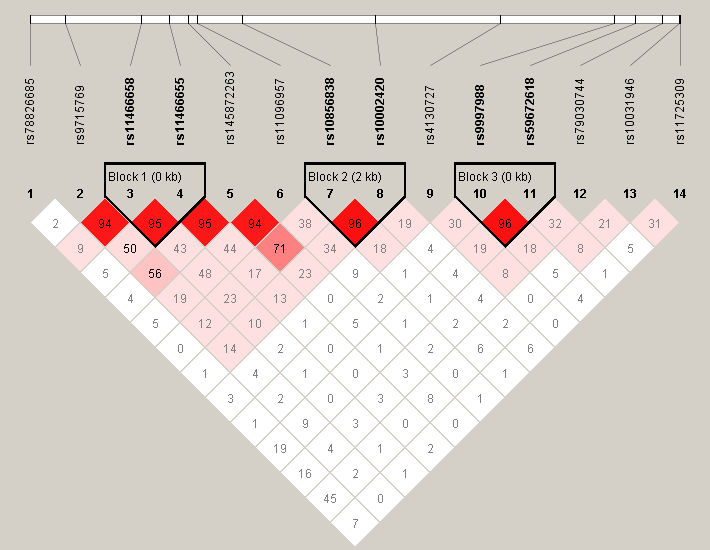


Supplemental Figure S2. LD structure of SNPs in TLR10. Values of D’ were indicated in each cell. D’ were also used as color scheme of this plot.


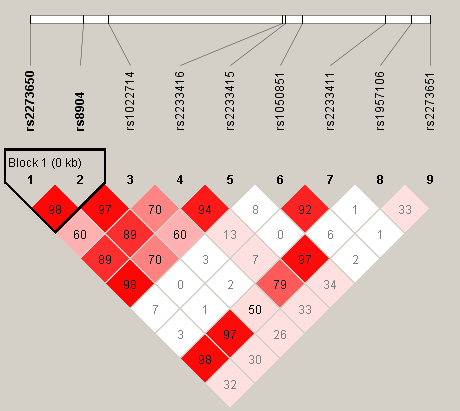


Supplemental Figure S3. LD structure of SNPs in *NFKBIA*. Values of D’ were indicated in each cell. D’ were also used as color scheme of this plot.
